# Application of Raman Spectroscopy to Rapid Discrimination of Autochthonous Lactic Acid Bacteria Isolated from Goat Cheese

**DOI:** 10.3390/cimb48050497

**Published:** 2026-05-11

**Authors:** Ana Yanina Bustos, Juan José Carol Paz, Jorge Nicolás Gómez, Ana Estela Ledesma

**Affiliations:** 1 Centro de Investigación en Biofísica Aplicada y Alimentos (CIBAAL-UNSE-CONICET), Universidad Nacional de Santiago del Estero (UNSE), RN 9, km 1125, Santiago del Estero 4206, Argentina; abustos@uspt.edu.ar (A.Y.B.); nicolasgoib@gmail.com (J.N.G.); 2 Facultad de Agronomía y Agroindustrias (FAyA), Universidad Nacional de Santiago del Estero (UNSE), Av. Belgrano (s) 1912, Santiago del Estero 4200, Argentina; 3 Facultad de Humanidades, Ciencias Sociales y de la Salud (FHCSyS), Universidad Nacional de Santiago del Estero (UNSE), Av. Belgrano (s) 2180, Santiago del Estero 4200, Argentina; 4 Departamento Académico de Química, Facultad de Ciencias Exactas y Tecnologías (FCEyT), Universidad Nacional de Santiago del Estero (UNSE), Av. Belgrano (s) 1912, Santiago del Estero 4200, Argentina

**Keywords:** Raman spectroscopy, lactic acid bacteria, chemometrics analysis, strain-level discrimination

## Abstract

The rapid characterization of lactic acid bacteria (LAB) with probiotic and technological properties is crucial for functional food design. In this study, fourteen LAB strains belonging to the species *Lactiplantibacillus* (*L.*) *plantarum*, *Lentilactobacillus* (*L.*) *parabuchneri*, and *Leuconostoc* (*L.*) *mesenteroides* were differentiated using Raman spectroscopy. By integrating Principal Component Analysis (PCA) and Linear Discriminant Analysis (LDA), we achieved a clear inter-generic separation while simultaneously enabling the intra-specific grouping of *L. plantarum* strains. Our results demonstrate that the Raman spectral fingerprint, coupled with supervised chemometric models, successfully categorized the strains into three distinct clusters based on their macromolecular profiles. Specifically, the analysis provided high-resolution differentiation between genera and, more importantly, allowed for the fine-scale clustering of diverse *L. plantarum* isolates. This highlights Raman spectroscopy as a robust, non-destructive tool for the rapid identification and taxonomic classification of LAB, offering a high-throughput alternative to traditional molecular methods for strain-level discrimination.

## 1. Introduction

LAB play a main role in the production of fermented products, due to their wide range of phylogenetic and functional diversity [1]. Moreover, some strains of LAB are widely used to produce functional foods because they are generally recognized as safe (GRAS) by the U.S. Food and Drug Administration, and offer numerous benefits for human health, such as stimulation of the immune system, production of antimicrobial substances, and promotion of energy homeostasis [2]. In addition, many of them tolerate the conditions of the gastrointestinal tract and are suitable for technological and industrial processes [3]. Therefore, the detection and characterization of new LAB strains with technological or probiotic properties is of great interest to the food industry for the development of new functional food products [4].

Raman spectroscopy has emerged as a promising analytical technique for microbial characterization due to several advantages over conventional identification methods such as Polymerase Chain Reaction (PCR) and MALDI-TOF mass spectrometry. Unlike PCR-based approaches, which rely on nucleic acid extraction and amplification to detect specific genetic sequences [5], Raman spectroscopy provides a holistic biochemical “fingerprint” of the cell, capturing information from proteins, lipids, and carbohydrates simultaneously. In comparison with MALDI-TOF mass spectrometry, which typically requires matrix application and sample preparation from cultured colonies [6], Raman spectroscopy can often be performed with minimal sample preparation and without chemical reagents, allowing rapid and non-destructive analysis. Furthermore, this technique enables the study of microorganisms at the single-cell level, preserving the metabolic state of the sample and facilitating real-time monitoring of microbial dynamics [7]. In Raman spectroscopy, the sample is illuminated with an incident laser, scattering the light with the vibrations of the molecular bonds, providing a direct biochemical fingerprint of microbial cells based on information from the bonds of nucleic acids, proteins, carbohydrates, and lipids that define the cell type identity and allow the discrimination and classification of microorganisms at the individual cell level [8,9,10,11]. Also, Raman spectroscopy does not require any previous knowledge of the samples under study and can offer patterns and groupings. Due to the complexity of the single-cell Raman spectrum, this analysis is usually accompanied by chemometric methods such as Principal Component Analysis (PCA) and Clusters Analysis (CA), among others [12]. The combination of these techniques not only facilitates the separation of different microorganisms but also provides abundant information on the composition of the cells studied.

Raman spectroscopy allows the monitoring of cellular states at the single-cell level, including viable but non-culturable probiotic bacteria [9], and enables the discrimination between intact and damaged cells during food processing [12].

Surface-Enhanced Raman Scattering (SERS) further expands the capabilities of Raman spectroscopy by enabling differentiation of bacteria at the genus, species, and strain levels, although it requires highly rigorous and precise experimental procedures [13]. The use of different SERS substrates plays a key role in improving bacterial discrimination. For example, silver nanoparticles (AgNPs) have been employed as SERS substrates to classify and identify LAB strains based on their spectral characteristics [14,15]. Additionally, Fe_3_O_4_ magnetic nanomaterials have been used in combination with SERS to separate and purify LAB and yeast at concentrations of 10^2^–10^4^ CFU/mL [16], while AuNPs@4-MBA substrates enable rapid identification of mixed LAB species [17]. More recently, the integration of SERS with machine learning approaches has demonstrated the ability to identify LAB at both species and subspecies levels [18,19].

Our working group recently isolated LAB strains from artisanal goat cheese with probiotic potential and proteolytic activity that were used to produce functional fermented goat milk [20]. Of these, 70% belong to the genus *Lactiplantibacillus*, while the rest were identified as belonging to the genera *Lentilactobacillus* and *Leuconostoc*. Species within the genus *Lactiplantibacillus*—notably *L. plantarum*—are of considerable technological and probiotic relevance due to their metabolic versatility, stress tolerance, and capacity to colonize diverse ecological niches. Genomically, *L. plantarum* exhibits pronounced intraspecies genetic heterogeneity, characterized by a large and flexible pan-genome, frequent horizontal gene transfer, and strain-specific gene clusters associated with carbohydrate utilization and environmental adaptation—features that underpin its ecological plasticity and functional diversity [21]. *L. parabuchneri* holds technological significance in dairy systems, particularly in cheese ripening, where it influences flavor development through proteolytic and amino acid catabolic activities, although certain strains may contribute to histamine accumulation [22]. Likewise, *L. mesenteroides* play a pivotal role in heterofermentative processes in dairy and vegetable fermentations enhancing texture, aroma, and sensory complexity, and contributing to food preservation through its production of bacteriocins [23]. Given the relevant properties of these strains, it is imperative to have a rapid and sensitive technique for their discrimination and characterization.

The objective of this research was to assess the capacity of Raman spectroscopy and PCA-LDA to quickly differentiate *L. plantarum*, *L. parabuchneri*, and *L. mesenteroides*, including strain-level identification for *L. plantarum*. Our findings highlight the potential of this approach for monitoring LAB strains and their application in fermented food characterization.

## 2. Materials and Methods

### 2.1. Bacterial Stain and Growth Conditions

Fourteen LAB strains from our strain collection were used in this study [24]. All strains were stored at −20 °C in a medium containing 10% (*w*/*v*) skim milk, 0.5% (*w*/*v*) yeast extract, and 30% (*v*/*v*) glycerol [25]. Before use, strains were activated in Man, Rogosa and Sharpe (MRS) medium for 24 h at 37 °C under microaerophilic conditions and subcultured three times in a similar manner [26]. *L. parabuchneri* CB12 and CB14, and *L. mesenteroides* CB6, were previously identified by Sesin et al. [24] using mass spectrometry and/or molecular methods (Table 1). In contrast, strains CB1, CB2, CB3, CB5, CB8, CB9, CB10, CB11, CB13, CB16, and CB17 were designated as *Lactiplantibacillus plantarum*/*paraplantarum*/*pentosus* since ribosomal protein fingerprints and 16S rDNA sequences lack the resolution to discriminate between these three species, given their high sequence identity (99%). Therefore, in this work, differentiation between species was performed using multiplex PCR assay with *recA* primers, as described below.

#### Differentiation of Species of the Genus *Lactiplantibacillus*

The differentiation of *Lactiplantibacillus* species was performed by amplifying the *recA* gene as detailed in Torriani et al. [27]. Briefly, genomic DNA was extracted from LAB by alkaline lysis, and the *recA* gene was amplified by multiplex PCR assay. The reverse primer (pREV, 5′-TCGGGATTACCAAACATCAC-3′) was used in combination with three species-specific primers (planF, 5′-CCGTTTATGCGGAACACCTA-3′, pentF, 5′-CAGTGGCGCGGTTGATATC-3′, and paraF, 5′-GTCACAGGCATTACGAAAAC-3′). The PCR protocol consisted of with initial denaturation at 94 °C for 3 min, 30 cycles of denaturation at 94 °C (30 s), annealing at 56 °C (10 s), and elongation at 72 °C (30 s), and final extension at 72 °C for 5 min.

The resulting products were visualized by staining a 1.5% (*w*/*v*) agarose gel with GelRed (Merck, Darmstadt, Germany). Expected amplicon sizes are 318 bp for *L. plantarum*, 218 bp for *L. pentosus*, and 107 bp for *L. paraplantarum*.

### 2.2. Raman Spectra of LAB Strains

Stationary-phase cultures (16 h), grown in MRS broth at 37 °C, under microaerophilic conditions and without agitation, were harvested by centrifugation (12,000× *g*, 10 min, 4 °C) and washed twice with a sterile saline solution (0.8% *w*/*v* NaCl). The pellets were resuspended in the same volume of sodium phosphate buffer solution (50 mM, pH 7.2). Drops containing 10 μL of each bacteria suspension were deposited on a microscope slide. Spectra were acquired using a Confocal Raman Horiba LabRAM HR Evolution (Instituto de Bionanotecnología del NOA, INBIONATEC-UNSE-CONICET, Santiago del Estero, Argentina) equipped with a 532 nm laser excitation source with a total power of 18 mW. A single bacterial cell was brought into focus with use of a 100× objective. Each spectrum consisted of an average of 8 measurements per strain from at least 8 different cells with an exposure time of 5 s in the range 100–2000 cm^−1^. The Raman Processing Software (LabSpec6 6.6.2.7) was used to import and analyze the data. Savitzky–Golay filtering was used for the smoothening of the spectral data with a 13-point window size and a third-order polynomial. Polynomial and rubber band methods were applied for baseline correction of spectral data. To further reduce the variation, average spectra were normalized on the basis of the most intense peak appearing close to 980 cm^−1^ using the OriginPro 8.5 program (MicrocalTM, Northampton, MA, USA). At least two biological replicates of each assay were performed.

### 2.3. Chemometric Methods

The multivariate Raman spectral datasets that minimize the dimensionality of the data and maximize the variation among spectral features are further analyzed using chemometric analysis tools. Principal Component Analysis (PCA) and Linear Discriminant Analysis (LDA) were performed on the Raman spectra, considering all of them, to evaluate spectral differences between the microorganism’s species as reported elsewhere [28]. PCA was used to group bacteria based on similarities between their Raman spectra. This analysis generates a data structure in a space defined by principal components (PC) capable of revealing the differences between microorganisms [28]. The PCA was performed using the free software Raman Tool Set-64bits (http://ramantoolset.sourceforge.net, accessed on 11 February 2026). Cross-validation for the LDA discrimination was done from the spectra using the “fitcdiscr.m” MATLAB Version: 9.13.0 toolbox.

## 3. Results and Discussion

### 3.1. Identification of Lactiplantibacillus Strains

**Figure 1:**
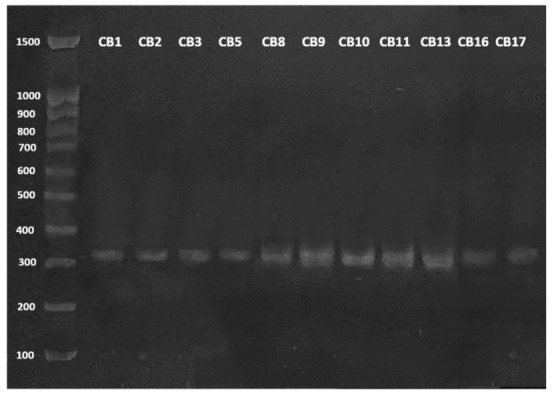
Species-specific identification of the *L. plantarum* group using multiplex PCR. Identification of *Lactiplantibacillus plantarum* strains by amplicon size (318 bp).

A total of fourteen LAB strains (Table 1) from our culture were collected and isolated from artisanal goat cheese and were used in this study [24]. According to Sesin et al. [24] *L. parabuchneri* CB12 and CB14, and *L. mesenteroides* CB6 were identified (Table 1), while the remaining strains were classified as *Lactiplantibacillus plantarum*/*paraplantarum*/*pentosus* group. To this end, a multiplex PCR assay targeting the *recA* gene was employed for the unequivocal identification of *L. plantarum*, *L. pentosus*, and *L. paraplantarum* in a single reaction. Based on this method, expected amplicon sizes were 318 bp, 218 bp, and 107 bp, respectively. As shown in Figure 1, all *Lactiplantibacillus* isolates yielded a product exceeding 300 bp, confirming their identification as *L. plantarum* (Table 1). In this regard, several authors have reported that the great metabolic versatility of these species makes them more ubiquitous than *L. paraplantarum* and *L. pentosus* species [29,30].

### 3.2. Raman Analysis and Bands Assignments for LAB Strains

Raman spectra were processed, normalized, and averaged for further analysis. Band assignments, detailed in Table 2, were based on established literature for the studied genera and species [14,15,16,17,18,19].

#### 3.2.1. *Leuconostoc mesenteroides* Strain

**Figure 2:**
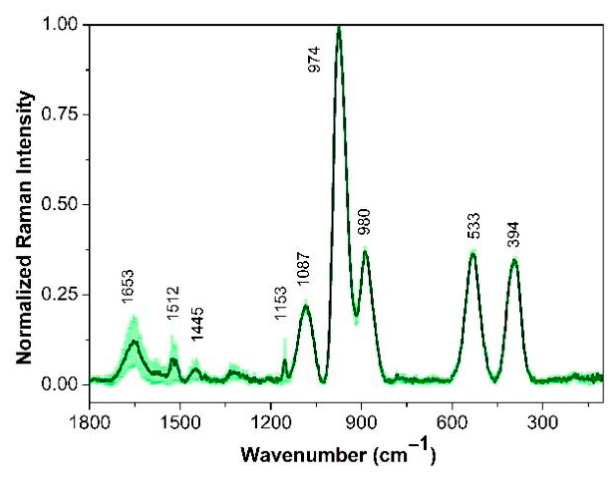
Raman spectra of *Leuconostoc mesenteroids* CB6. The mean Raman spectra is indicated by the solid line (green), and the standard deviations are represented by the shadow. Each Raman spectrum is normalized to its corresponding maximum peak close to 974 cm^−1^.

The average Raman spectrum of *L. mesenteroides* CB6 exhibited peaks of varying intensities (Figure 2). The spectral range analyzed aligns with that reported by Yamamoto et al. [31], which defines a molecular fingerprint for differentiating food-spoilage bacteria. Notably, the spectrum of the CB6 strain displayed a prominent, high-intensity peak at 974 cm^−1^ which was associated with the stretching vibration between carbon atoms that make up the carbon skeleton of proteins (960–980 cm^−1^) or carbohydrates (C-O-C) stretching, as was detailed by Vaitikiekunaite et al. [12] for *L. mesenteroides* species. Additional peaks showed a strong correlation with other reported bands for this microorganism assigned to protein vibrations (1653–1528 cm^−1^), nucleic acids (1087, 394 cm^−1^), and lipids (890 cm^−1^).

#### 3.2.2. *Lentilactobacillus parabuchneri* Strains

**Figure 3:**
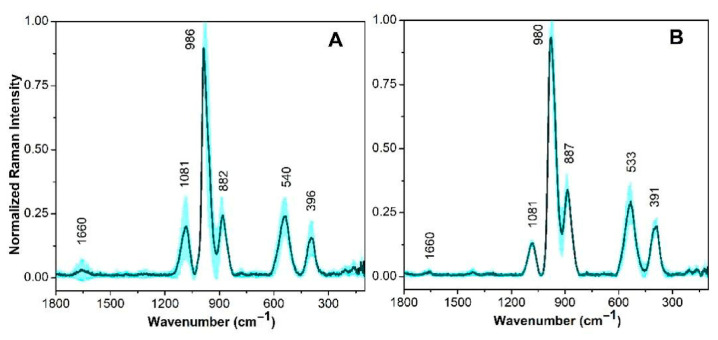
Raman spectra of *Lentilactobacillus parabuchneri* CB12 (**A**) and CB14 (**B**) strain. The mean Raman spectra is indicated by the solid line (cyan), and the standard deviations are represented by the shadow. Each Raman spectrum is normalized to its corresponding maximum peak close to 980 cm^−1^.

Figure 3 illustrates the spectral variance between *L. parabuchneri* strains CB12 and CB14. Major characteristic peaks in both strains were assigned to proteins (1660 and 533 cm^−1^), carbohydrates (980 cm^−1^), nucleic acids (1081 and 396 cm^−1^), and lipids (shoulder at 887 cm^−1^). These assignments align with previous reports for strain CB12 [32]. Furthermore, differences in the Amide I and Amide II bands, which exhibited significantly lower intensities compared to those of *L. mesenteroides* CB6, were reflected in the intensity ratios, contributing substantially to the accurate discrimination of these LAB species.

Raman characterization of *L. parabuchneri* spectra showed a strong correlation with Fourier Transform Infrared (FTIR) spectroscopy data reported by Bajrami et al. [32].

**Table 2:** Raman bands assignments for the three genera used in this study in the 1800–100 cm^−1^ region.

*L. mesenteroides*		*L. parabuchneri*		*L. plantarum*		Tentative Assignment
This Work	SERS [16]	This Work	FTIR [33]	This Work	Raman [15,19,21,22]	
1653	1644	1660	1640	1655–1662	1662	Amide I, Amide II (proteins)
				1553–1575	1582–1584	Adenine (DNA), C=C stretching vibration (benzone rings)
1528	1529					Asymmetric stretching (COO-) proteins
1512	1499					Phe
1445	1465	1416	1455	1412–1453	1447–1454	C-H bending (lipids)
1335	1327			1305–1335	1321–1396	Amide III (proteins)
			1238	1207–1247	1228–1236	Amide III (proteins)
1153	1163					Stretching (C-C),
1087	1096	1081	1086	1080–1198	1077–1099	PO_2_^−^ in nucleic acid, or stretching (P-O), stretching (C-C), lipids, N-C stretching (phospholipids), proteins, or C-C skeletal and C-O-C stretching, glycosidic link (carbohydrates)
974	956	980	970	976–993	958–1099	Carbohydrates, C-COO stretching, stretching (C-O) and (C-N) of proteins, stretching (C-C), stretching (C-O), skeletal proteins, phenilalanine
890	881	882		858–891	850–886	stretching (C-O-C), stretching (C-C), polysaccharides; symmetric stretching (C-C-N), lipids, proline, hydroxiproline, tyrosine, pririmidine rings
				779–784	729–785	PO_2_^−^ of DNA backbone, citocine, uracile, adenine, nucleic acids
				665–678	667–696	C-S stretching cysteine
533	522	540		532–369	554–604	C-C stretching (carbohydrates), S-S stretching (proteins)
394	412					Phosphate group or skeletal modes of CC,
	355	396		405		Phosphate group

Abbreviations: ν, stretching; PO_2_^−^, phosphate.

#### 3.2.3. *Lactiplantibacillus plantarum* Strains

Spectroscopic characterization of *L. plantarum* strains allowed for the differentiation of three distinct groups based on the degree of spectral overlap or peak compatibility. Differentiation between microorganisms of the same species is a fairly common phenomenon because this technique is highly susceptible to the physical state of the bacteria, its growth conditions, and the processing of samples prior to measurement. In fact, recently, a method based on signal-to-noise ratio of Raman spectra made it possible to identify and differentiate two strains of LAB of the same species but from different sources [19].

**Figure 4:**
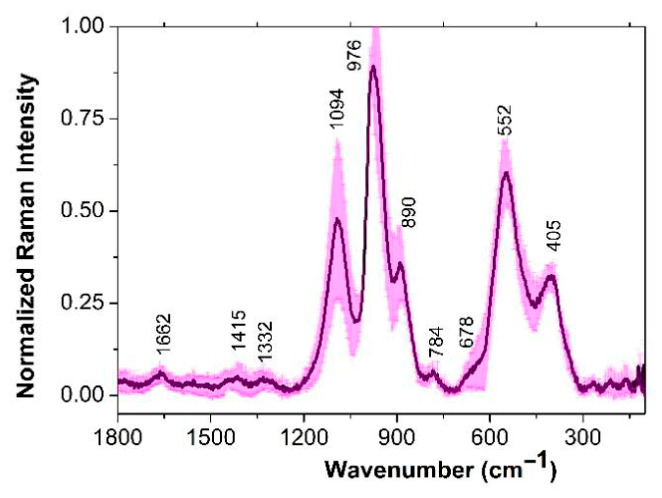
Raman spectra of *L. plantarum* CB2 strain. The mean Raman spectra is indicated by the solid line (magenta), and the standard deviations are represented by the shadow. Each Raman spectrum is normalized to its corresponding maximum peak close to 976 cm^−1^.

The first group comprised a single strain, *L. plantarum* CB2 (Figure 4), which exhibited characteristic bands at 1662 cm^−1^ (proteins), 1415 cm^−1^ (lipids), 1332 cm^−1^ (amino acids), 1094 cm^−1^ (PO_2_^−^ of nucleic acid), 976 cm^−1^ (carbohydrate), 784 cm^−1^ (DNA), 552 cm^−1^ (proteins), and 405 cm^−1^ (phosphate groups). Additionally, a shoulder at 678 cm^−1^ was attributed to cysteine residues. These peak positions are consistent with those reported for differentiating *L. plantarum* SR1 [19] and Z1 [18] from other species such as *Pediococcus* (*P.*) *pentosaceus*, *Lactococcus lactis* and *Saccharomyces cerevisiae*.

Notably, the band at 1094 cm^−1^ (PO_2_^−^ of nucleic acid) exhibited a blue shift of approximately 10 cm^−1^ compared to our *L. mesenteroides* and *L. parabuchneri strains* before mentioned. Furthermore, the intensity of this band, along with the protein-related band at 552 cm^−1^, was doubled in the CB2 strain. In this strain, DNA-associated phosphate bands were also localized at higher wavenumbers than those observed in the other LAB species studied.

**Figure 5:**
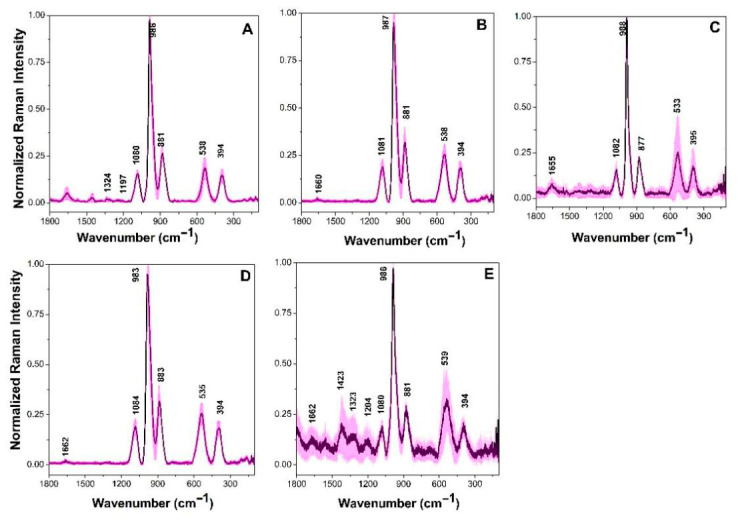
Averaged Raman spectra of *L. plantarum*. (**A**) CB8, (**B**) CB10, (**C**) CB11, (**D**) CB13 and (**E**) CB17. Magenta areas show spectral intensity variations in individual Raman shifts. Each Raman spectrum is normalized to its corresponding maximum peak close to 987 cm^−1^.

The second group comprised *L. plantarum* strains CB8, CB10, CB11, CB13 and CB17 (Figure 5). This grouping was primarily defined by the high degree of spectral overlap across five prominent peaks of similar intensity. A central peak at 986 cm^−1^ was attributed to the carbon skeleton vibrations of carbohydrates and proteins, as well as the carboxyl group of proteins [20,21]. This aligns with the peak at 985 cm^−1^ reported for the *L. plantarum* Y1 strain, which helped differentiate it from the *P. pentosaceus* Z1 strain [19]. Other characteristic peaks were identified at 536, 881, and 1081 cm^−1^, assigned to disulfide bonds in proteins, lipids and nucleic acid vibrations, respectively [16,17]. Additionally, a peak at approximately 394 cm^−1^ was observed; while not previously reported for *L. plantarum*, a similar signal in *L. mesenteroides* RSG7 has been associated with phosphate group vibrations [34].

**Figure 6:**
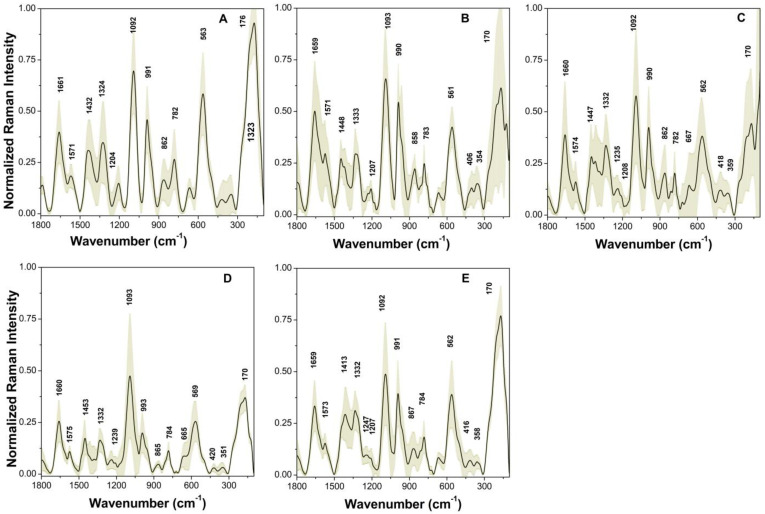
Averaged Raman spectra of *L. plantarum.* (**A**) CB1, (**B**) CB3, (**C**) CB5, (**D**) CB9 and (**E**) CB16. Green areas show spectral intensity variations in individual Raman shifts. Each Raman spectrum is normalized to its corresponding maximum peak.

A third group of *L. plantarum* strains is presented in Figure 6. Notably, these strains exhibited significant differences spectrally, both among themselves and compared to the previously described *L. plantarum* groups. The most prominent peaks were observed at 1659, 1093, 991, and 563 cm^−1^, with additional notable bands at 1579, 1440, 1333, 1207, 862, 774, 670, 418, and 360 cm^−1^ (assigned in Table 2). The relative intensity of the peak at 1093 cm^−1^ was markedly higher than in the other strains analyzed. Furthermore, a distinctive band at 1579 cm^−1^ was attributed to amino acid vibrations, specifically the contribution of tryptophan [8]. These high-resolution spectra are consistent with recent SERS reports for *L. plantarum*, where Raman shifts correlate with typical vibrational modes of DNA/RNA, proteins, and lipids [22].

### 3.3. Spectral Biomarkers and Intracellular Composition

The intensities of the characteristic peaks attributed to DNA (1094 cm^−1^), carbohydrates (976 cm^−1^), and lipids (890 cm^−1^) were normalized to distinguish between strain groups. Carbohydrate/DNA, DNA/lipid, and carbohydrate/lipid ratios were calculated, as shown in Table 3. For *L. plantarum* CB2 (Group 1), the carbohydrate/DNA ratio was approximately 2, while the DNA/lipid and carbohydrate/lipid ratios were 1.3 and 2.5, respectively. In contrast, Group 2 (CB8, CB10, CB11, CB13, and CB17) exhibited higher carbohydrate/DNA values (ranging from 5 to 6), with DNA/lipid ratios around 0.6 and carbohydrate/lipid ratios near 4. Group 3 strains showed a carbohydrate/lipid ratio below 1; It is worth noting that these strains showed high DNA signals and low carbohydrate peak intensities. For *L. parabuchneri* (CB12 and CB14), these ratios were 7, 0.4, and 3, respectively. Finally, *L. mesenteroides* yielded values of 4.5 (carbohydrate/DNA), 0.6 (DNA/lipid), and 2.7 (carbohydrate/lipid). These spectral variations could reflect differences in that main macromolecules signals among the species, consistent with existing literature, where reports showed that Raman band intensities can be used to calculate ratiometric markers (I_830_/I_810_, I_1126_/I_1100_, I_1340_/I_1440_, I_1207_/I_1240_ and I_1580_/I_1440_) of biomolecules such as DNA, proteins and lipids to identify the growth phase of bacteria, regardless of culture condition [34].

### 3.4. Principal Component Analysis of Raman Spectra

**Figure 7:**
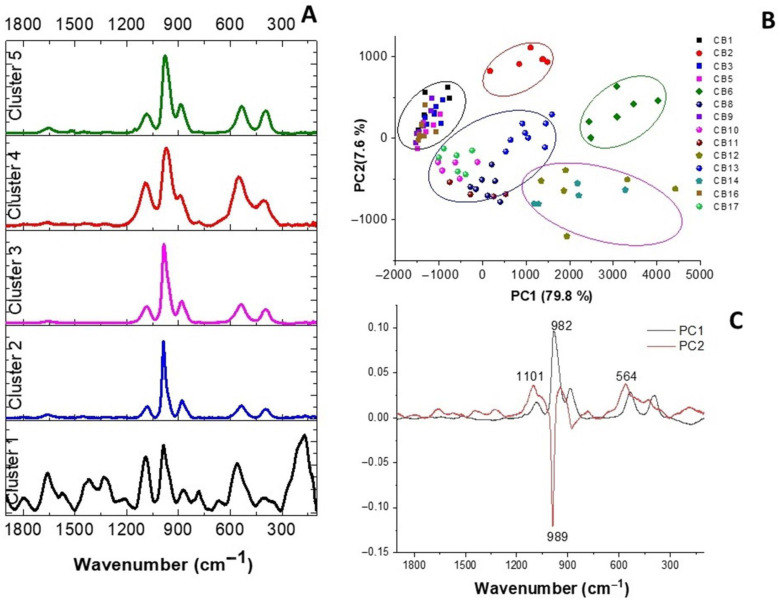
(**A**) Average spectra for the five-cluster segmentation in 100–1900 cm^−1^ spectral region. (**B**) Plot of PC1 vs. PC2 to discriminate all LAB. The colored circles indicate each grouping of strains. (**C**) Depicts the PC1 and PC2 loadings respectively.

K-means clustering was performed on the Raman spectra to elucidate the chemical relationships between the different strains. Figure 7A displays the average spectra for the five cell clusters identified. These spectral profiles reveal variations in relative band intensities, reflecting differences in cellular composition, such as protein and lipid content. To develop a classification routine based on Raman characteristics (capturing inter-group differences and intra-group similarities), all normalized spectra were subjected to PCA. This approach identified five distinct regions where data points share similar chemometric features (Figure 7B); the circles in Figure 7B highlight the groups exhibiting the most significant spectral variations.

PC1 accounts for 79.8% of the explained variance and enables the discrimination of five groups: Group 1 (strains CB1, CB3, CB5, CB9; black), Group 2 (strain CB2; red), Group 3 (strains CB8, CB10, CB11, CB13, CB17; blue), Group 4 (strains CB12, CB14; magenta), and Group 5 (strain CB6; green). These clusters are distributed across the four quadrants of the scores plot. While Group 1 is positioned on the positive side of the PC1 axis, Groups 3, 4, and 5 are on the negative side. Group 2 is distributed across both. These results demonstrate that PC1 effectively discriminates between *L. plantarum*, *L. parabuchneri*, and *L. mesenteroides*. PC2 (7.6% variance) depicts the intra-species differentiation within *L. plantarum* (Groups 1 and 2) and *L. parabuchneri*.

The PCA scores plot confirms a clear separation between genera and individual strains. To investigate the drivers of this separation, the loading plots were analyzed (Figure 7C). The PC1 loading plot revealed that variations in the carbohydrate band (~982 cm^−1^) and the carbohydrate/protein ratio (564 cm^−1^) were prominent features for discriminating *L. parabuchneri*, *L. mesenteroides*, and *L. plantarum* Group 1 from the remaining LAB. In contrast, the PC2 loading plot is more complex, showing negative features responsible for the differences within *L. plantarum* strains. Notably, *L. parabuchneri* can be further discriminated by a carbohydrate peak close to 986 cm^−1^, a feature also prominent in other bacterial genera studied in this work.

**Figure 8:**
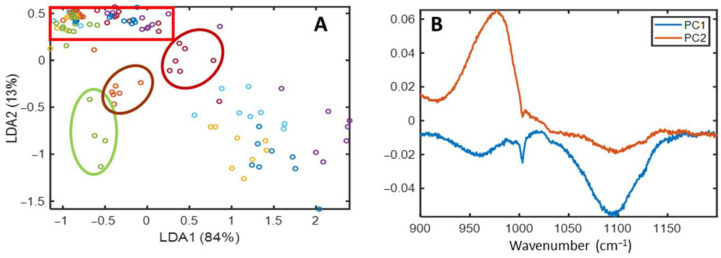
The LDA results of the Raman spectra of the fourteen microorganisms in 900–1200 cm^−1^ region (**A**). CB6 (green circle), CB2 (brown circle) and CB9 (red circle). PC1 and PC2 loadings, for this spectral region, respectively (**B**).

After dimensionality reduction via PCA, the dataset was analyzed using supervised methods; specifically, a LDA was performed in the 900–1200 cm^−1^ range using the resulting PC scores. Figure 8 illustrates the score plots for the PCA-LDA model, based on LD1 (84%) and LD2 (13%). These plots show a clear differentiation between the CB6 (green)**,** CB2 (brown), and CB9 (red) datasets, effectively separating *L. mesenteroides* (CB6) from the *L. plantarum* strains (CB2 and CB9), as well as distinguishing the latter two from each other.

In Figure 8B, the negative peaks along PC1 correspond to Raman signals from nucleic acids and cellular carbohydrates. Meanwhile, the positive peaks along PC2 align with spectral signals related to carbohydrate components, as previously discussed. These results demonstrate that combining LDA with the 900–1200 cm^−1^ spectral range enables the rapid and accurate identification of *L. mesenteroides* and *L. plantarum*. Furthermore, this approach reinforces the differentiation within the *L. plantarum* group, matching results reported by more complex Raman methodologies [19].

Cross-validation for the LDA discrimination of CB2, CB6 and CB9 was done and the resulting confusion matrix is shown in Table 4, with a single misclassified sample, leading to the following figures of merit (Table 5).

Our study successfully differentiated *L. plantarum* strains into three distinct groups. These results indicate that combining PCA and LDA statistical analyses enables the rapid and accurate identification of diverse LAB types. Similarly, Ren et al. [18] identified nine LAB species and subspecies—including this genus—using single-cell Raman spectroscopy.

## 4. Conclusions

Our results show that Raman spectroscopy, combined with PCA-LDA chemometric analysis, is a promising and rapid tool for the characterization and discrimination of the genera *Lentilactobacillus*, *Leuconostoc*, and *Lactiplantibacillus*. Furthermore, based on their unique biochemical profiles, different species of *L. plantarum* were successfully grouped. Unlike traditional molecular techniques, this method offers a non-destructive, reagent-free alternative with a high sensitivity for strain-level discrimination, making it an ideal candidate for high-throughput screening in the development of functional foods.

## Figures and Tables

**Table 1:** LAB used in this study identified.

Strains	Mass Spectrometry *	Molecular Identification *	Multiplex PCR Assay Based on *recA* gene ^Ұ^
CB1	*Lactiplantibacillus plantarum*/*paraplantarum*/*pentosus*	*Lactiplantibacillus plantarum*/*paraplantarum*/*pentosus*	*Lactiplantibacillus plantarum*
CB2	*Lactiplantibacillus plantarum*/*paraplantarum*/*pentosus*	*Lactiplantibacillus plantarum*/*paraplantarum*/*pentosus*	*Lactiplantibacillus plantarum*
CB3	*Lactiplantibacillus plantarum*/*paraplantarum*/*pentosus*	*Lactiplantibacillus plantarum*/*paraplantarum*/*pentosus*	*Lactiplantibacillus plantarum*
CB5	*Lactiplantibacillus plantarum*/*paraplantarum*/*pentosus*	*Lactiplantibacillus plantarum*/*paraplantarum*/*pentosus*	*Lactiplantibacillus plantarum*
CB6	*Leuconostoc mesenteroides*	*Leuconostoc mesenteroides*	
CB8	*Lactiplantibacillus plantarum*/*paraplantarum*/*pentosus*	*Lactiplantibacillus plantarum*/*paraplantarum*/*pentosus*	*Lactiplantibacillus plantarum*
CB9	*Lactiplantibacillus plantarum*/*paraplantarum*/*pentosus*	*Lactiplantibacillus plantarum*/*paraplantarum*/*pentosus*	*Lactiplantibacillus plantarum*
CB10	*Lactiplantibacillus plantarum*/*paraplantarum*/*pentosus*	*Lactiplantibacillus plantarum*/*paraplantarum*/*pentosus*	*Lactiplantibacillus plantarum*
CB11	*Lactiplantibacillus plantarum*/*paraplantarum*/*pentosus*	*Lactiplantibacillus plantarum*/*paraplantarum*/*pentosus*	*Lactiplantibacillus plantarum*
CB12	*Lentilactobacillus parabuchneri*	*Lentilactobacillus parabuchneri*	
CB13	*Lactiplantibacillus plantarum*/*paraplantarum*/*pentosus*	*Lactiplantibacillus plantarum*/*paraplantarum*/*pentosus*	*Lactiplantibacillus plantarum*
CB14	*Lentilactobacillus parabuchneri*	*Lentilactobacillus parabuchneri*	
CB16	*Lactiplantibacillus plantarum*/*paraplantarum*/*pentosus*	*Lactiplantibacillus plantarum*/*paraplantarum*/*pentosus*	*Lactiplantibacillus plantarum*
CB17	*Lactiplantibacillus plantarum*/*paraplantarum*/*pentosus*	*Lactiplantibacillus plantarum*/*paraplantarum*/*pentosus*	*Lactiplantibacillus plantarum*

* Bacteria identified in the work of Sesin et al. [24], ^Ұ^ Species identified in the present work.

**Table 3:** Raman band ratio of major cellular components including carbohydrates/DNA (976/1094 cm^−1^), DNA/lipids (1094/890 cm^−1^) and carbohydrate/lipids (976/890 cm^−1^) for each strain groups.

Microorganisms	Strain	976/1094 Ratio	1094/890 Ratio	976/890 Ratio
*L. plantarum* (Group 1)	CB2	1.9	1.4	2.4
*L. plantarum* (Group 2)	CB8	5.7	0.6	3.8
	CB10	6.1	0.7	4
	CB11	6	0.7	4.3
	CB13	5	0.6	3
	CB17	5.1	0.6	3.3
*L. plantarum* (Group 3)	CB1	0.6	4.9	3.2
	CB3	0.8	3.1	2.5
	CB5	0.9	2.8	2.1
	CB9	0.4	10	4.2
	CB16	0.8	3.7	3
*L. parabuchneri*	CB12	4.5	0.8	4.5
	CB14	7.1	0.4	2.7
*L. mesenteroids*	CB6	4.5	0.6	2.7

**Table 4:** Cross-validation for the LDA discrimination of CB2, CB6 and CB9.

Microorganisms	CB2	CB6	CB9
CB2	7	1	0
CB6	1	7	0
CB9	0	0	8

**Table 5:** The accuracy or correct discrimination rate for all classes = 100 × (correctly classified)/(total), sensitivity = % of samples of class i, correctly classified as i, specificity = % of samples of class ≠ i, correctly classified as not i, and efficiency = (sensitivity × specificity)1/2.

Figure of Merit		Global	
Accuracy		91.7%	
	CB2	CB6	CB9
Sensitivity	87.5%	87.5%	100%
Specificity	93.8%	93.8%	87.5%
Efficiency	90.6%	90.6%	93.5%

## Data Availability

The original contributions presented in this study are included in the article. Further inquiries can be directed to the corresponding author.
